# Complexity, cofactors, and the failure of AIDS policy in Africa

**DOI:** 10.1186/1758-2652-12-12

**Published:** 2009-07-10

**Authors:** Eileen Stillwaggon

**Affiliations:** 1 Department of Economics, Gettysburg College, Gettysburg PA 17325, USA

## Abstract

Global AIDS policy still treats HIV as an exceptional case, abstracting from the context in which infection occurs. Policy is based on a simplistic theory of HIV causation, and evaluated using outdated tools of health economics. Recent calls for a health systems strategy – preventing and treating HIV within a programme of comprehensive health care – have not yet influenced the silo approach of AIDS policy.

Evidence continues to accumulate, showing that multiple factors, such as malnutrition, malaria and helminthes, increase the risk of sexual and vertical transmission of HIV. Moreover, complementary interventions that reduce viral load, improve immune response, and interrupt pathways of transmission could increase the effectiveness of antiretroviral drugs and other tools of AIDS policy.

In health economics, the omission of estimates of increasing returns generated by disease or treatment synergies biases cost-effectiveness analysis against multiple, yet inexpensive, interventions. Current tools of cost-effectiveness analysis only identify local maxima in a complex landscape, and can play, at best, a marginal role in the epidemic, especially where it is already generalized.

Cost-effectiveness analyses for HIV that are based on the wrong epidemiological model can generate Type III errors: we get precise answers to the wrong questions about how to intervene. To control the epidemic, AIDS policy needs to utilize an epidemiological model that reflects the interactions of biological as well as behavioural variables that determine the course of HIV epidemics around the world. Cost-effectiveness analysis can benefit from using economic concepts of externalities and increasing returns to incorporate disease interactions and beneficial treatment spillovers for coinfections in HIV-prevention policy.

## Introduction

Over the past 25 years, global AIDS prevention policy has remained largely isolated from mainstream epidemiology, which recognizes that epidemics arise from the interaction of multiple biological characteristics of the host, pathogen and environment. The result has been an implicit (and often explicit) theory of HIV causation that treats AIDS as a special case, unrelated to the context of malnutrition or parasitic and infectious disease in which the epidemic flourishes.

Health economics uses tools such as cost-effectiveness analysis to evaluate alternative interventions for prevention and treatment. Just as AIDS policy is isolated from the conventional understanding of disease interactions, health economics has remained isolated from an expanding toolkit in other fields of economics that recognize interaction and incorporate externalities and increasing returns. Thus it is poorly equipped to evaluate interventions that have spillover benefits, such as when treating one disease or condition improves the efficacy of interventions to prevent or treat other conditions.

This article discusses how the limitations of health economics reinforce the errors caused by a simplistic theory of HIV causation and artificially isolate AIDS programming from other health-promotion priorities. It suggests better integration of complex models of epidemiology with economic models of increasing returns to develop more effective AIDS interventions through a broader health-systems approach.

Although HIV is sexually transmitted in probably the majority of cases in poor populations in developing countries, exclusive focus on the proximate cause of infection (sexual contact) does not provide an explanation for the divergence in incidence among populations. Sexual behaviour, of course, is important in determining individual risk, but differences in sexual behaviour between countries do not correlate with differences in HIV prevalence or incidence. Numerous empirical studies demonstrate that rich countries have higher rates of most risky behaviours – early initiation of sex, short-term concurrent relationships, unprotected sex, multiple partners and premarital sex – that are not matched by high rates of HIV [1-11]. Long-term concurrent relationships may be more common in parts of sub-Saharan Africa [12,13], but national rates of concurrency do not correlate with rates of HIV [14].

Without coinfections or other conditions that raise per-contact transmission rates, there is no evidence that African concurrency or networks are more risky than sexual networks elsewhere. The appropriate comparison for Africa is not with a hypothetical case of no sexual networks, but with real sexual networks, such as are reported on North American university campuses, with extensive short-term concurrency and high rates of sexually transmitted infections (STIs) [3,6,9], and yet low rates of HIV.

Prevention policy for HIV/AIDS is not evidence-based. In spite of accumulating survey evidence that national or regional rates of HIV do not correlate with rates of risky behaviours [1,15,16], AIDS policy still emphasizes sexual behaviour to the exclusion of other factors that influence individual and national vulnerability to HIV. To devise effective prevention strategies, we need to understand why HIV spreads at different rates in different populations.

## What is driving the spread of HIV in poor populations?

A growing body of scientific literature demonstrates that host and ecological factors play an important role in determining an individual's vulnerability to HIV infection and the contagiousness of HIV-infected partners (and mothers). Sexual contact with an infected person represents only a necessary, but not sufficient, condition for infection through sex. Similarly, being born to an HIV-infected mother does not always lead to infection of the infant. Vertical transmission, before introduction of maternal prophylaxis, ranged from 14% of infants of HIV-infected mothers in Europe to 40% in sub-Saharan Africa [17].

Moreover, in the absence of other factors, transmission of HIV in industrialized countries has been insufficient to maintain a heterosexual epidemic. In the United States and western Europe, among otherwise healthy adults, transmission from females to males will occur in about one out of 1000 contacts, and from males to females, about once in 500 contacts [17]. Clearly, other factors play a role in determining individual infection and divergence in incidence of HIV in different regions.

In the 1990s, STIs were recognized as potential cofactors for HIV transmission, and STI treatment has been included in some HIV-prevention programmes [18]. Some skeptics cite the Rakai trial [19], which did not seem to confirm the role of STIs in HIV transmission, but that trial had serious flaws, including the absence of a true control group. Rakai's "control" group was treated with deworming medication and a vitamin/mineral supplement, with results not significantly different from treating STIs. (For additional discussion and sources, see [5].)

Later it was recognized that antiretroviral therapy (ART), by reducing viral load, could reduce transmission [20]. More recently, male circumcision has been added to prevention programmes. But AIDS policy does not yet address the widespread nutritional, parasitic and infectious conditions that can act as cofactors of HIV transmission. The following section discusses prevalence of cofactors primarily in sub-Saharan Africa because HIV is much higher there than elsewhere. The divergence in HIV epidemics in different world regions suggests a complex etiology for generalized epidemics and the need for systemic solutions that solve multiple problems simultaneously.

### Nutritional deficiencies

From 1988 to 1998, when nascent or concentrated AIDS epidemics developed into generalized epidemics in sub-Saharan Africa, 30% of the population of the region was malnourished [21]. Malnutrition increases vulnerability to infectious and parasitic diseases generally, and it increases HIV viral load and viral shedding, thereby increasing sexual and vertical transmission of HIV [5,22-30].

### Malaria

More than 90% of acute malaria infections worldwide occur in tropical Africa. Africa accounts for the majority of malaria deaths, including about 3000 deaths per day of children under the age of five. Survivors suffer chronic immune activation through repeated reinfection, increasing individual susceptibility of HIV-negative persons [31]. Malaria increases HIV viral load as much as 10-fold, increasing contagiousness of HIV-infected persons and affecting the dynamics of the epidemic at the population level [5,32-37]. Individuals in malaria-endemic areas have a higher probability of sexual contact with persons who are infected with both malaria and HIV, and who thus have high viral load. Models of malaria-HIV interaction estimate a three-fold increase in HIV transmission in malaria-endemic populations and increased malaria transmission due to HIV coinfection [32].

### Filariasis and geohelminthes

Lymphatic filariasis afflicts over 40 million people in sub-Saharan Africa. Prevalence is increasing in Africa because breeding areas for mosquitoes proliferate with unplanned urban growth [38]. Helminthic infections (various kinds of worms) are widespread in developing countries and virtually ubiquitous in shanty towns and rural communities. Worldwide, nearly 1.5 billion people are infected with ascariasis, 1.3 billion with hookworm, and more than 1 billion with trichuriasis [39]. Lymphatic filariasis and soil-transmitted helminthes have also been shown to suppress immune response in HIV-negative persons and increase viral load in HIV-infected persons, affecting individual transmission and population dynamics [5,39-44]. A recent double-blind, controlled trial found that treating ascariasis in HIV-infected persons results in a statistically significant increase in CD4 counts [45]. That suggests that a simple, inexpensive (2 US cents) and effective deworming medication (albendazole) could allow HIV-infected people to postpone ART. On an individual and population basis, the benefits of postponing first-line ART are substantial.

### Schistosomiasis

Urinary schistosomiasis (*S. hematobium*) afflicts almost 200 million people in sub-Saharan Africa [46] and acts as a co-factor of HIV transmission in much the same way as do STIs. Acquired in contaminated lakes and streams, worms and ova of *S. hematobium *infect the reproductive tracts of both men and women. They create lesions, which are open portals for HIV, and inflammation of the genital area, which makes HIV transmission more efficient [5,47-50]. In Zimbabwe, researchers found that genital lesions of schistosomiasis increased HIV risk in women three-fold compared to women in the same communities without genital schistosomiasis [51]. Furthermore, reports from developing countries indicate that neglecting to treat schistosomiasis, worms and malaria contributes to failure of ART.

As we acquire new information about HIV transmission, we have to make sure we are asking the right questions in each regional context. Viral load is an important factor in determining the risk of infection [52], and recent studies have concluded that the majority of new infections are transmitted by persons who were themselves recently infected and thus have high viral load [53]. That is probably true in poor populations as well.

In coinfected persons in poor populations, however, the burden of malnutrition, parasites and infectious diseases also increases viral load, not just within the first weeks of HIV infection, but over the lifetime of the infected person. Malarial episodes, for example, increase viral load not just during febrile periods, but for seven weeks afterwards [32,36,54] and people in endemic zones are repeatedly infected.

We can expect to see a flurry of studies on how best to reach newly infected persons with elevated viral load. That will be very useful, of course, but we also need to consider the extended periods of elevated viral load in persons with malaria and other coinfections.

In light of the conventional epidemiological understanding of disease synergies and the evidence that interactions with specific parasites and infections increase vulnerability to and contagiousness of HIV, the exclusively behavioural focus of AIDS policy reveals a very simplistic notion of disease causation.

## Limitations of the behavioural paradigm

The primary focus of HIV-prevention policy and the principal targets of spending continue to be various strategies for changing sexual behaviour. Substantial money and effort have been expended, thus far without success, on vaccine and microbicide development, but those efforts also reflect a focus on proximate cause.

That the major political debates on AIDS policy have revolved around promoting abstinence or providing condoms attests to the behavioural focus of HIV-prevention policy. Comprehensive lists of standard interventions [55-57] name various behaviour-change strategies for sexual and needle-sharing behaviour and strategies for reducing mother-to-child transmission. The additions of STI treatment and male circumcision may be important steps, but AIDS programming is still restricted to factors fairly obviously connected to a proximate cause, sexual contact.

While AIDS discourse does address social and economic factors, such as stigma, gender roles and poverty, it is only to the extent that such factors influence risky sexual behaviours, closing off other useful lines of inquiry. Gender analysis of AIDS includes important issues, such as violence and inheritance practices, but not the mundane risks of gendered household tasks, such as washing clothes or gathering reeds in rivers and lakes, which affect susceptibility to HIV through genital lesions of schistosomiasis [58].

Studies of higher HIV prevalence among fisherfolk and carwashers (who work standing in lake water) presume unobserved sexual networks at the lakeshore. They fail to consider that people who work in fresh water in Africa have high rates of schistosomiasis, which increases HIV transmission. We cannot examine AIDS in a laboratory where social and economic factors affect only sexual behaviour. People have sex and bear children in a context of everyday risks – disease vectors, contaminated water, food insecurity and job hazards – that make every sexual contact and every birth more risky in poor countries.

### The standard model of HIV in policy documents

In the policy literature, HIV-prevention interventions are justified on the basis of an extremely simplistic model of HIV transmission. The standard models for sexual transmission of HIV used by multilateral and bilateral donors (for example, AVERT, GOALS and STDSIM [59-61]) do not incorporate what is known about the complexity of HIV transmission. They usually assume a universal dose-response, given in a constant per-contact transmission risk. The core of the standard model is as follows:

where *I *is probability of sexual infection, *N *is the number of partners, *P *is the prevalence rate in the population, and *T *is the per-contact transmission risk, which is assumed to be the same for every population.

Each of the models is different, but the core variables included in each can be represented with this simple equation. AVERT includes number of sex partners, number of sex acts, prevalence of STIs, condom use and related variables. GOALS includes various behaviour-change interventions and can include blood-safety interventions.

Because the models include only population characteristics related to sexual behaviour or sexual health, they explain incidence of infection through the behavioural variable, *N*, or number of partners. In circular fashion, the only policy conclusions that derive from this equation favour behavioural interventions. STDSIM, for example, could incorporate schistosomiasis along with STIs, but it does not. Omitting cofactors leads to biased estimations.

STDSIM was used to evaluate whether rates of male circumcision could explain the differences in rates of HIV in the Four Cities Study [15], allowing for variation in other risk factors, such as frequenting commercial sex workers. The model was successfully fitted to the data, except for the case of Kisumu, Kenya, leading the authors to reject the behavioural data the men reported. Had they included prevalence of schistosomiasis for Kisumu, which is on Lake Victoria, their model might have predicted HIV in Kisumu better, and they would not have had to conclude that the men were lying about their sexual behaviour [62].

The implicit assumption underlying these models and most HIV-prevention strategies is that differences in sexual behaviour, represented by *N*, explain differences in HIV rates, although the preponderance of evidence shows no correlation at the country level between rates of various sexual behaviours and rates of HIV.

*P*, prevalence, is a misleadingly simple concept. Transmission dynamics are influenced not just by the proportion of the population that is HIV-infected, but by the infectiousness of each person infected. *P*, thus, should not be a number, but an array of numbers representing the proportion of the population infected at each level of viral load. It matters a great deal at the individual and at the population level what the population viral load is.

In this equation, prevalence, *P*, represents the probability of a sexual contact being HIV infected. But what matters more is the probability that a random sexual partner from that population has a viral load above the level at which transmission is likely to occur [52,63]. Consequently, our understanding of the spread of HIV, in sub-Saharan Africa in particular, would be improved if we estimated *P *as an array disaggregated by level of viral load. And our prevention of transmission would be enhanced by interventions that reduce viral load in infected partners and mothers.

Transmission risk, *T*, should include per-contact risk of infection for the HIV-negative person (vulnerability) and per-contact risk of transmission for the HIV-infected person (contagiousness). Both vulnerability and contagiousness could be increased by infection with helminthes, malaria, malnutrition, tuberculosis, STIs and schistosomiasis. (To avoid double-counting the enhanced contagiousness of the HIV-infected person, we would include here only factors not captured in the estimate of viral load in the array of *P*. Genital sores of schistosomiasis or STIs in either partner would be included here, for example.)

Similarly, a new model for vertical transmission should include characteristics of mother and infant, including anemia and other nutritional deficiencies, geohelminth exposure, schistosomiasis and STIs. Epidemic models for poor countries should also include primary transmission by contaminated medical instruments and blood. Even if such medical transmission produces only the 5% to 10% of primary infections conservatively estimated by UNAIDS [64], that is an underestimated source of primary infection for women and infants in particular, since both are more likely to undergo invasive medical procedures than are men [65,66].

The standard model, with a scalar *P *and a constant *T*, assumes that one risk fits all individuals and all populations, abstracting from almost all the important biological variation between rich and poor, and temperate and tropical populations. Modifying *P *and *T *helps, but it does not begin to model the complex interactions among conditions or to estimate the effect of non-linearities in the impact of one or more conditions on others. We would need more fully specified models to do that, although it may not be possible or mathematically meaningful to aggregate all conditions across all individuals.

The point is that if we use a model that assumes one risk fits all, as do most models used in AIDS policy, we cannot explain the global distribution of HIV and AIDS, and we cannot generate useful prevention policies for different regions. By cataloguing all the endemic conditions that are known to influence the spread of HIV in poor populations, and accounting for the disease synergies, we can attempt to design HIV programmes that address the differential risk of multi-burdened populations.

That does not, however, mean that we should postpone treatment for widespread, debilitating conditions until we have the perfect model. We already have plenty of evidence that treating STIs, helminthes, malaria and malnutrition are good things in themselves. The only barriers to addressing those problems have been a lack of political will and flawed economic models.

## Complexity and HIV

We need new ways of thinking about HIV causation, and here, I outline one way to begin. The AIDS epidemic, like most epidemics, is a complex, contingent process. In complex adaptive (or contingent) systems, even small differences in initial conditions can result in widely different outcomes, and sudden or rapid change can produce bifurcations, or changes in trajectory. Edward Lorenz observed such results in the 1960s, when his modelling of weather conditions calculated at three decimal places produced widely different forecasts from his calculations at seven decimal places [67].

As epidemics unfold as complex, contingent processes, both the exposures (sexual contacts for HIV) and the infections themselves result from multiple, interacting causes. Sequential iterations produce new trajectories that are determined (not random), but unpredictable at the outset. In different countries and different regions, relevant conditions of hosts, environment and sometimes the pathogen can differ not just by small amounts, but by several orders of magnitude.

In the United States and Europe, for example, malaria and schistosomiasis are extremely rare, whereas in Africa the number of malaria cases per year exceeds 25% of the population in 27 countries [68] and the number of schistosomiasis cases exceeds 25% of the population in 22 countries [69]. Differences in the burden of other parasitic diseases are similarly vast.

It is simplistic to assume that a large event, such as a generalized HIV epidemic, results from a single large cause, a lone gunman. It is more likely that large events are the result of the synergistic effect of multiple causes, each of which may show slight variation between regions. The divergence in HIV incidence between rich and poor countries and between temperate and tropical areas is affected by the interplay of malaria, STIs, helminthes, filariases, anemia, vitamin-A deficiency and many other factors, for which the differences between rich and poor populations are great.

Moreover, even between western and southern Africa, the relative weights of each of those factors differ, although they are not differences in order of magnitude. As in weather patterns, it is quite plausible that very small differences in initial conditions of one or several factors can result in very different outcomes in incidence of diseases with multiple, interacting determinants.

### Boolean networks

One way to visualize how AIDS epidemics behave as complex systems with interacting variables is to use Boolean networks, employed by some biologists [70]. In a system with *N *elements (for example, diseases or environmental factors), some of those elements interact and are called inputs, designated as *K*. In simple systems, of *K *= 2 or *K *= 3, stable outcomes can be expected. When every variable is connected to every other variable – a so-called *K *= *N *network – outcomes are said to be completely random [70,71], although perhaps it should be said that they are determined but unpredictable.

A Boolean network seems to model HIV effectively. In developed countries, it may be that *K *= 2 or *K *= 3, and incidence would therefore be stable and predictable; this has generally been the case in western Europe and North America. In poor populations in tropical regions, with many interacting variables (malaria, malnutrition, worms, etc.), *K *approaches *N*, and epidemics are unstable and unpredictable. The greater the number of factors, the more sensitive are the outcomes (epidemic trajectories) to initial conditions.

As Kauffman observed in reference to other *K *= *N *systems, minimal changes typically cause extensive damage – alterations in the activity patterns – almost immediately [70] (p. 81). This approach might be extremely useful when applied to HIV, considering the divergent evolutions of HIV epidemics in different populations. No other credible explanation has been offered for the near-explosive growth of HIV in southern Africa. And no single variable can explain the differences between regions, be it male circumcision, labour migration patterns, local sexual practices or parasite burden.

Clearly, if *K *= *N *or nearly so, policy makers must work with a reduced form of the model. They have to choose the most significant coinfections for interventions. But to make policy that is relevant to a real-world epidemic, and even to recognize the most significant coinfections, they need to bear in mind that such complex interactions of multiple factors determine the diverging trajectories in different regions.

The good news is that while disease interactions can accelerate epidemics, they also provide multiple entry points to interrupt transmission. Many of those opportunities, such as providing clean water and sanitation and deworming, are much more policy sensitive than sexual behaviour, and they have multiple beneficial effects.

## The poverty of economics

Ignoring interacting and multiple-level variables has generated an inadequate theory of disease causation to inform AIDS policy. That problem is reinforced because of the limitations of health economics, and cost-effectiveness analysis in particular, in evaluating complex interventions. Unlike other fields of economics, health economics has not been drawn into the exploration of complexity, non-linearity and multiple equilibria.

Since disease interactions have non-linear effects, multiple outcomes are not only possible, but quite likely in disease dynamics. But recognition of non-linearities is rare in health economics. (In a search of all articles published in *Health Economics*, *Health Policy and Planning*, and *Journal of Health Economics *in the past 10 years, I found only three articles that made any reference to interaction variables or non-linearities. None was based on a model of biological interaction.)

Clearly, policy makers must employ some method of evaluating interventions, and the principle of cost-effectiveness analysis is valid. Cost-effectiveness analysis, however, is best used when there are identical outcomes to alternative treatments or when it is easy to measure a single objective (outcome) of the intervention [72].

In an epidemic with multiple, interacting causes, it is difficult to define interventions with identical outcomes or to evaluate treatments with only one kind of benefit. The use of simple cost-effectiveness analysis appears to validate the superiority of single-input interventions because, as it is currently employed, it cannot measure the benefits of programmes with heterogeneous or diffuse benefits, unanticipated spillover benefits, or benefits that take some time to appear. With few notable exceptions [73], they fail to recognize increasing returns (decreasing costs) or other non-linearities in interventions.

## Increasing returns: economics in the real world

The general equilibrium model that has dominated economics for more than a century assumes negative feedbacks (decreasing returns) that lead to a unique, stable equilibrium under perfect competition.

Since the 1980s, economists have attempted to model positive feedbacks in growth theory, trade theory and other fields [74-77]. Positive feedbacks (increasing returns) generally provide a better description of actual economic conditions, especially of the past 200 years. They do not lead to a unique, stable equilibrium (are not boundary defending), but instead can have multiple possible outcomes (misleadingly called equilibria). Health economics, however, is based on the conventional economic model of the early twentieth century that assumes decreasing or constant returns (increasing or constant costs), although that is rarely stated explicitly.

Increasing returns can occur for various reasons. The simplest case is that of scale economies, where the fixed costs of a clinic, for example, are spread over a larger number of patients, and the marginal cost of additional patients is negligible. A second type is economies of scope, where fixed costs are spread over more services, and additional services are virtually costless. Where economies of scope are present, modelling a number of services together would give more valid estimates of cost effectiveness than requiring each service to be justified independently, as is more generally the case in cost-effectiveness analysis.

Health economists tend to focus on short-run diminishing returns, rather than scale economies, and belabour the increasing cost of bringing treatment to ever more remote villages. But that is generally not the situation in the field.

In reality, tens of thousands of people in poor countries with generalized HIV epidemics die without any treatment even though they live in close proximity to health facilities. Reaching them does not entail rising costs per person. Many clinics can still expand the range of services and the number of people served with decreasing average cost. As word spreads about ART and treatment of coinfections, such as ascariasis, schistosomiasis and malaria, outreach can be self-sustaining. And as the number of people receiving treatment grows, there are also population benefits (herd effects) for both prevention and treatment, which lower future costs.

Cost-effectiveness analysis and health economics generally would be more useful if they incorporated more health information. They need to be interdisciplinary, using economic tools but reflecting the underlying biological complexity of conditions they hope to address. To integrate the economics and the biology, I would add to the conventional economic notions of economies of scale and scope two categories of biologically based increasing returns arising from disease interactions.

One is the positive treatment spillover within the individual. For example, treatment for cofactor conditions, including worms, malnutrition and malaria, makes HIV prevention and ART more effective for each individual. The second biologically driven cause of increasing returns is the population effect. Treatment for HIV, helminthes, TB and malaria (among other things) reduces transmission in the population by reducing HIV viral load, parasite burden and infectiousness of TB.

To calculate accurately the costs of delivering services for a population, we have to include the population spillovers (benefits) that reduce viral load, transmission, and subsequent costs of prevention and treatment. If we do not calculate conventional economies of scale and scope *and *intra-individual treatment benefits (such as deworming for HIV) *and *externalities (population effects), we seriously underestimate the benefits of complementary interventions, and we allocate resources improperly. Ultimately, we need to broaden the scope of what we consider treatment outcomes in calculating benefits of programmes that affect multiple sectors. Deworming, for example, not only improves health, it also improves school attendance and cognitive development, but a health sector evaluation might not include those benefits.

The result of using simplistic cost-effectiveness analysis that can compare only very similar interventions is that the tool can identify only local maxima (minima). But the landscape of HIV transmission is very complex. If we only want to advise policy makers on whether it is better to hand out condoms in a community centre or in a factory, or hand out condoms with or without lollipops as an inducement, the current methods are adequate. If, however, we want to reduce the significant relative risks in poor environments (compared to rich environments), those risk factors must be included in the models, and the benefits of complementary interventions have to be calculated.

The generation of local maxima is essentially a Type III error – we get very precise, and even correct, answers to the wrong questions [78]. But it will not matter very much if one approach to condom distribution is marginally better than another if the larger reason that HIV spreads so rapidly in poor populations is the prevalence of endemic parasitic and infectious diseases.

## Conclusion

HIV epidemics, like other complex systems, are influenced by multiple factors. The issue is not that sexual behaviour is unimportant. It is that behaviour explains so little about why poor people get sick, especially in tropical areas with little access to safe medical care, clean water, sanitation and good housing to protect them from disease vectors.

Standard HIV-prevention policies, and thus cost-effectiveness analyses evaluating those interventions, have overlooked complementary investments for treating coinfections. Treatment for TB, schistosomiasis, malaria, malnutrition and helminthes is relatively inexpensive, highly effective, and essential for improving immune status in HIV-negative persons and decreasing viral load in HIV-infected persons.

Such investments are not a diversion of funds from HIV prevention; they are necessary complements. Deworming is safe, effective and easily dosed; it generates positive externalities [79]; and it might also prevent the failure of first-line ART – at a cost of as little as 2 US cents per person. The cost of moving to second-line therapy will far exceed the cost of treating coinfections.

We need cost-effectiveness tools that reflect complexity and attempt to measure the costs of multiple inputs distributed over multiple outputs in which interactions play a prominent role. Those tools would recognize conventional economies of scale and scope, which are extensive in multi-purpose programmes, as well as biological externalities, both intra-individual and population-wide, that, if exploited, reduce overall cost. With those tools, we can achieve our goal of healthy individuals in healthy populations, rather than chasing after one virus, one person at a time.

## Competing interests

The author declares that she has no competing interests.

## Author's information

Eileen Stillwaggon is Professor of Economics and Harold G. Evans-Eisenhower Professor at Gettysburg College.
